# Increasing Incidence of Zoonotic Visceral Leishmaniasis on Crete, Greece

**DOI:** 10.3201/eid1506.071666

**Published:** 2009-06

**Authors:** Maria Antoniou, Ippokratis Messaritakis, Vasiliki Christodoulou, Ioanna Ascoksilaki, Nikos Kanavakis, Andrew J. Sutton, Connor Carson, Orin Courtenay

**Affiliations:** University of Crete, Heraklion, Greece (M. Antoniou, I. Messaritakis, V. Christodoulou); Veterinary Laboratory of Heraklion, Heraklion (I. Ascoksilai); Veterinary Services, Sitia, Greece (N. Kanavakis); University of Warwick, Coventry, UK (A.J. Sutton, C. Carson, O. Courtenay)

**Keywords:** Leishmania infantum, zoonotic visceral leishmaniasis, canine leishmaniasis, incidence, models, zoonoses, parasites, Crete, Greece, dispatch

## Abstract

To determine whether the incidence of canine leishmaniasis has increased on Crete, Greece, we fitted infection models to serodiagnostic records of 8,848 dog samples for 1990–2006. Models predicted that seroprevalence has increased 2.4% (95% confidence interval 1.61%–3.51%) per year and that incidence has increased 2.2- to 3.8-fold over this 17-year period.

Zoonotic visceral leishmaniasis (ZVL) caused by *Leishmania infantum* is a disease of humans and domestic dogs (the reservoir) transmitted by phlebotomine sandflies. According to the World Health Organization (*1*), ZVL was first recorded on Crete in 1907, after which it featured prominently in medical literature as a serious public health problem. In Chania, Crete, the annual incidence in the 1930s was 50 cases/30,000 population (*2*), and 21% of 1,115 dogs were positive for ZVL by the formol-gel serologic test; 30% of those were symptomatic (*1*).

After World War II, use of DDT against malaria vectors and focal destruction of *Leishmania* spp.–infected dogs is thought to have reduced ZVL on Crete (*3*). Sandflies were not found in villages systematically sprayed during 1946–1949 compared with unsprayed villages (*4*). During 1951–1975, only 33 alleged human ZVL cases were recorded on Crete (*5*), and reports of canine ZVL were scanty. In 1983 a serosurvey of 72 stray dogs in Chania identified only 1 infected dog (*1*).

By the late 1980s, *Phlebotomus neglectus*, the putative vector of *L*. *infantum* on Crete (*6*), was abundant in stone walls inside and outside villages around Heraklion (*7*). During 1999–2004, *P*. *neglectus* was found in abundance in human dwellings and rural locations (hollows in olive trees and near rodent burrows) (*8*). Since 1991, 38 persons who came to hospitals in Heraklion were confirmed as having cases of ZVL. Today, canine infection is confirmed throughout the island (M. Antoniou, unpub. data), and seroprevalences (30%–40%) are some of the highest reported in Europe. These accounts anecdotally suggest that the incidence of ZVL has increased on Crete during the past decade, which, if so, would be relevant to the public and veterinary health sectors.

## The Study

We statistically examined diagnostic records of 8,848 dogs sampled in the eastern Crete districts of Lasithi and Heraklion during 1990–2006. Data were supplied by the Faculty of Medicine, University of Crete (n = 1,205 dogs from 97 villages tested by using an indirect immunofluorescent antibody test [IFAT], cutoff titer 160, during 1999–2006, accompanied by demographic and geographic records); and by the Ministry of Agriculture Serology Laboratory, Heraklion (n = 7,643 dogs tested by using an IFAT, cutoff titer 200, during 1999–2005, but without accompanying records). Samples were collected by veterinarians in private practice from any dog initially brought to their clinic for any reason, or by government veterinarians for any dog encountered, irrespective of clinical condition, during prearranged visits to villages. Numbers of villages and dogs sampled in any year depended on available resources at the time.

To reduce potential sampling bias, we first tested serologic data from 6 villages (located 9–45 km apart) consistently sampled annually during 1999–2006 (n = 744 dogs). The age-adjusted annual prevalence increased significantly with sample year (odds ratio [OR] 1.18, 95% confidence interval [CI] 1.089–1.271, p<0.001) and showed no significant differences in slope or intercept (by using robust standard errors) compared with data from 91 less consistently sampled villages with demographic records (n = 461 dogs; slope × village group interaction OR 0.91, 95% CI 0.800–1.023, p not significant; intercepts OR 5.35, 95% CI 0.902–3.688, p not significant).

These combined datasets were then compared with crude prevalence data for 1990–2005 (n = 7,643 dogs) calculated from the ministry records; no difference was detected in prevalence slopes (slope × data source interaction OR 1.06, 95% CI 0.995–1.30, p not significant; Figure). In univariable or multivariable logistic regression that controlled for dog age and clustering on villages (1999–2006, n = 1,205 dogs), no statistical confounding of the probability of a dog being seropositive was attributed to dog use (companion, guard, or hunting dog), sex, crude habitat type, or village altitude (p>0.215; see Technical Appendix Table). The final fit of seroprevalence against time was significant (OR 1.24, 95% CI 1.221–1.264, p<0.001), and the linearized difference in model prevalence (and binomial confidence limits) over the 17-year study equated to a mean prevalence increase of –ln(1 – 0.321)/16 = 2.4% (95% CI 1.61%–3.51%) per year.

To assess the change in infection incidence, our principal aim, we used 3 standard epidemiologic models (*9**–**11*; Technical Appendix) to calculate infection rates accounting for time, dog age, and potential loss of infection. The first method (model 1) used the cross-sectional age-prevalence data (IFAT cutoff titer 160), in which the proportion of seropositive dogs in each age class is fitted by varying the rates of infection and recovery. A second method (model 2) used these same data to describe the infection rate as it varied with both time and age until reaching the best fit. The third method (model 3) estimated the infection rate from longitudinal data of previously unexposed dogs <12 months of age that were followed up during 1 transmission season.

Results from the 3 models were consistent (Tables 1, 2) and showed a relative increase in the mean infection rate estimated to be 2.20–3.78-fold higher during 2005–2006 than during 1999–2000. The models differed in approach and age of dogs considered by necessity of the model, number of estimated parameters, or model reduction. Inclusion of a parameter describing loss of infection (Table 1, model 1) did not significantly lower the infection rate estimates as might be expected compared with a single parameter (Table 1, model 2) or longitudinal (Table 2, model 3) model, both of which identified younger (<2 years of age) dogs to be at substantially greater risk for infection (p<0.0001).

**Table 1:** Variation in incidence over time estimated from cross-sectional data for 1,205 dogs with accompanying demographic records, Crete, Greece, 1990–2006*

Period	Model 1					Model 2		No. dogs
	Incidence/mo	95% CI	Loss of infection/mo	95% CI		Incidence/mo	95% CI	
1999–2000	0.016	0.0107–0.0206	0.045	0.0260–0.0645		0.015	0.0093–0.0213	237
2001–2002	0.029	0.0114–0.0455	0.071	0.0201–0.1211		0.023	0.0166–0.0298	219
2003–2004	0.030	0.0216–0.0381	0.049	0.0320–0.0667		0.029	0.0221–0.0367	401
2005–2006	0.059	0.0233–0.0946	0.106	0.0383–0.17430		0.032	0.0205–0.0477	348

*Only model 1 is designed to estimate loss of infection (serorecovery). CI, confidence interval. A full description of these models is available in the Technical Appendix.

**Table 2:** Variation in incidence over time estimated from longitudinal data for 179 dogs with accompanying demographic records, Crete, Greece, 1990–2006*

Period	Model 3		
	No positive/ no. tested	Incidence/ mo	95% CI
1999–2000	6/56	0.014	0.0061–0.0280
2001–2002	7/30	0.033	0.0153–0.0612
2003–2004	12/56	0.030	0.0172–0.0490
2005–2006	10/37	0.039	0.0210–0.0670

*CI, confidence interval. A full description of this model is available in the Technical Appendix.

## Conclusions

The potential contribution of any improvements in diagnostic test sensitivity or vigilance to the increasing incidence of ZVL infection is unclear. The difference in cutoff titers between data sources minimally shifted the absolute prevalence values, but not relative prevalence slopes, with time (Figure). Any loss of infection with age (Table 1, model 1) could result from nonmutually exclusive biologic processes including recovery from infection, death, or reduced past exposure (*9**,**11*). The latter possibility is unlikely on Crete because of the higher risk identified in young dogs in all biannual periods. Disproportionate numbers of deaths of seropositive dogs is not suggested by a decline in ZVL clinical signs in older dogs in this study (data not shown) or elsewhere (*11**,**12*). Loss of detectable *Leishmania*-specific antibody is the more likely explanation because the observed rates of serorecovery are not dissimilar to those (e.g., 0.062/month) estimated by cohort studies elsewhere (*12*).

**Figure:**
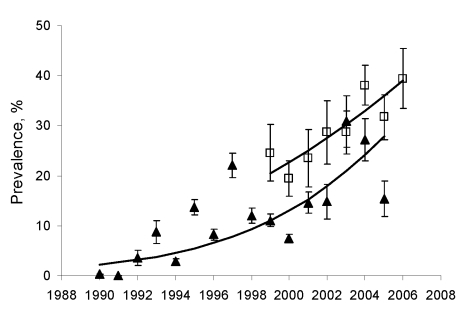
Annual seroprevalence of zoonotic visceral leishmaniasis in dogs on Crete, Greece, 1990–2006. Shown are logistic fits of age-adjusted prevalences (line and squares) for dogs from 97 villages (indirect immunofluoresecent antibody test [IFAT] cutoff titer 160) and crude seroprevalences (line and triangles) calculated from records of the Veterinary Laboratory of Heraklion, Crete, Hellenic Ministry of Rural Development and Food (www.minagric.gr) (IFAT cutoff titer 200). Binomial standard error bars are shown.

Actual infection rates are likely to be higher than those shown here because IFAT sensitivity is <100%. Similarly, absolute prevalences, particularly low values for 1990–1991, should be treated with caution because the official leishmaniasis control program on Crete (1984–1995) began before this period when infection was presumably sufficient on the island to warrant intervention. The intervention comprised elimination of IFAT-seropositive dogs (cutoff titer 400) but did not include insecticide spraying (DDT spraying ceased in 1950; V. Chatzistefanou, pers. comm.).

The continual increase in canine seroprevalence during the latter part of the intervention (Figure) suggests that the culling policy was unsuccessful in reducing transmission; likely reasons for the low efficacy of dog culling in other leishmaniasis-endemic regions have been described (*13*). Officially, destruction of seropositive dogs is still required today unless the owner agrees to veterinary treatment of the dog or to keep the dog under sandfly-proof netting. However, there is no current policy on Crete to combat vectors. We conclude that the results of our study are consistent with a postwar reemergence and current increasing incidence of ZVL infection on Crete.

## Supplementary Material

Technical Appendix Analysis of Demographic and Environmental Associations with Canine Prevalence of Zoonotic Visceral Leishmaniasis
