# Supplementary material for: Increasing Incidence of Zoonotic Visceral Leishmaniasis on Crete, Greece
Source: Emerg Infect Dis. 2009 Jun;15(6):932–4. doi: 10.3201/eid1506.071666 (PMC2727332; doi:10.3201/eid1506.071666)
Supplement: Technical Appendix — Analysis of Demographic and Environmental Associations with Canine Prevalence of Zoonotic Visceral Leishmaniasis [file 07-1666_Techapp-s1.pdf]

Increasing Incidence of Zoonotic Visceral Leishmaniasis on Crete, Greece

Technical Appendix

Analysis of Demographic and Environmental Associations with Canine Prevalence of Zoonotic Visceral Leishmaniasis

The multivariate full logistic model to test potential effects of demographic and environmental conditions on canine prevalence is shown in the Technical Appendix Table.

Technical Appendix Table. Demographic and environmental factors potentially affecting the likelihood of infection in dogs on Crete, Greece, 1990–2006*

Variable	Odds ratio (95% confidence interval)	z statistic	p value	No.
Age, y†	0.03 (–0.004 to 0.065)	1.74	0.081	1,205
Dog use				
Hunter	Baseline			850
Pet	0.82 (0.579–1.171)	–1.08	0.279	183
Guard	0.79 (0.512–1.223)	–1.06	0.291	172
Sex				
Male	Baseline			485
Female	1.01 (0.756–1.338)	0.04	0.969	720
Vegetation type‡				
A	Baseline	–0.42	0.676	682
B	0.73 (0.165–3.222)	–0.42	0.676	24
C	1.40 (0.822–2.382)	1.24	0.215	159
D	1.13 (0.694–1.829)	0.48	0.630	114
E	0.95 (0.571–1.588)	–0.19	0.850	226
Elevation, m				
>0–50	Baseline			648
>50–100	0.72 (0.371–1.383)	–0.99	0.320	103
>100–500	1.23 (0.752–2.014)	0.83	0.408	325
>500–880	0.98 (0.561–1.724)	–0.06	0.954	129

*Statistics for the full multivariate logistic regression model included values for baseline categorical levels, controlling for effects of dog age (also shown), and clustering observations by village (where dog lived).
†Noncategorical variable. Statistics represent regression coefficient, not odds ratio.
‡Vegetation types according to the Corine biotype and habitat classification scheme (http://biodiversity-chm.eea.europa.eu/information/document/F1088156525). A, urban fabrics; B, nonirrigated arable land; C, olive groves and fruit and berry plantations; D, complex cultivation patterns; E, land largely occupied by agriculture with areas of natural vegetation including natural grassland and *Sclerophyllous* spp.).

The goodness-of-fit of the full model was assessed by using the Pearson statistic (χ^2_{419} = 426.95, p>0.384) and the Hosmer-Lemeshow statistic on a collapsed number of groups (n = 10)

on the basis of the deciles of the predicted logits ($\chi^2 = 7.28$, df 8, p>0.507). Both statistics were in agreement that the number of infected dogs predicted by the model was not significantly different from the observed number. None of the variables tested were significant in univariable analysis.

Epidemiologic Models Used to Calculate Infection Rates

Model 1

Cross-sectional age-prevalence (indirect immunofluorescence antibody cutoff titer 160) was fitted to a 2-parameter model by finding the proportion of seropositive dogs p(*a*), at mean age *a*, from

$$p(a) = \frac{\lambda}{\lambda + \rho}\left(1 - e^{-(\lambda+\rho)a}\right)$$

by varying the cumulative rate of infection (λ) and recovery rate (ρ) where $\lambda/(\lambda + \rho)$ is the asymptotic proportion of positive animals. The model was fitted by maximum likelihood methods according to Williams and Dye (*1*) (results in Table 1 in the main text, model 1). Age prevalences were calculated for biannual periods by using mean ages of standardized age-class intervals 0–12 >12–24, >24–36, >36–60, and >60 months selected to give approximately equal numbers of dogs per age class. The model assumes that rates of infection and recovery are constant with age, that the population is homogeneously exposed, and that seroconversion immediately follows exposure. Loss of seropositivity (infection) in this model may indicate a loss of detectable antibody titer with age (time), disproportionately high death rates among seropositive dogs, or changes in population exposure. Under the model assumptions, the average age (in months) of patent infection is equivalent to 1/λ, and the average duration of seropositivity detected by the test is 1/ρ.

Model 2

According to methods previously described (*2*), the cumulative rate of infection in 1998 by age *a* is described by the function *f*(*a*)

$$f(a) = z + \frac{(u - z)}{1 + (e^{(a-w)})^{v}}$$

where *u*, *z*, *w*, and *v* are model constants capturing changes in the cumulative rate of infection with age. *f*(*a*) describes all infections in previous years and is estimated for infection rates for more recent years (1999–2006). No attempt has been made to reduce the parameters describing this function because the priority is to ensure sufficient flexibility and a good fit to the data. The rate of infection from 1999 onwards is then modeled as the product of a function describing its trend over time *g*(*t*) and a function describing its trend with age *h*(*a*)

$$\lambda(t,a) = g(t)h(a)$$

To standardize results *h*(*0*) is fixed to equal 1. Both *g*(*t*) and *h*(*a*) are parameterized on piecewise constant functions. The model was fitted to the binomial prevalence data (infected, not infected) by using maximum likelihood. The most parsimonious reduced model (results in Table 1 in the main text, model 2) was achieved by comparing model deviance ($d_1 = 122.1$) with a nested model in which variation in the infection rate was held constant across ages (deviance $d_2 = 180.3$), showing the extra parameter describing dog age (2 categories: ≤ 2 and >2 years) to be highly significant ($d_2 - d_1 = 58.2$, df 1, $p<0.0001$). Thus, this parameter was retained in the final model.

Model 3

As previously described (*3*), the rate of infection λ was estimated from the proportion (p) of previously unexposed dogs ≤12 months of age (n = 179) born before the transmission season that seroconverted to positive during a follow-up exposure period of t = 8 months, where $p = 1 - e^{(-\lambda t)}$ (results in Table 2, model 3). Incidence values were expressed in months.

References

1. Williams BG, Dye C. Maximum likelihood for parasitologists. Parasitol Today. 1994;10:489–93. PubMed DOI: 10.1016/0169-4758(94)90163-5

2. Sutton AJ, Gay NJ, Edmunds WJ, Hope VD, Gill ON, Hickman M. Modelling the force of infection for hepatitis B and hepatitis C in injecting drug users in England and Wales. BMC Infect Dis. 2006;6:93. PubMed DOI: 10.1186/1471-2334-6-93

3. Courtenay O, MacDonald DW, Lainson R, Shaw JJ, Dye C. Epidemiology of canine leishmaniasis: a comparative serological study of dogs and foxes in Amazon Brazil. Parasitology. 1994;109:273–9. PubMed
